# Analgesic use during pregnancy and risk of infant leukaemia: A Children's Oncology Group study

**DOI:** 10.1038/sj.bjc.6606046

**Published:** 2010-12-14

**Authors:** S Ognjanovic, C Blair, L G Spector, L L Robison, M Roesler, J A Ross

**Affiliations:** 1 Division of Pediatric Epidemiology and Clinical Research, University of Minnesota, 420 Delaware Street SE, MMC 422, Minneapolis, MN 55455, USA; 2 Masonic Cancer Center, University of Minnesota, 425 E River Road, 554 MCRB, Minneapolis, MN 55455, USA; 3 Department of Epidemiology and Cancer Control, St Jude Children's Research Hospital, 262 Danny Thomas Place, MS-735, Memphis, TN 38105, USA

**Keywords:** epidemiology, infants, leukaemia, analgesics

## Abstract

**Background:**

Infant leukaemia is likely initiated *in utero*.

**Methods:**

We examined whether analgesic use during pregnancy was associated with risk by completing telephone interviews of the mothers of 441 infant leukaemia cases and 323 frequency-matched controls, using unconditional logistic regression.

**Results:**

With the exception of a reduced risk for infant acute myeloid leukaemias with non-aspirin non-steroidal anti-inflammatory drugs (NSAID) use early in pregnancy (odds ratios=0.60; confidence intervals: 0.37–0.97), no statistically significant associations were observed for aspirin, non-aspirin NSAIDs, or acetaminophen use in early pregnancy or after knowledge of pregnancy.

**Conclusion:**

Overall, analgesic use during pregnancy was not significantly associated with the risk of infant leukaemia.

Infant leukaemia, diagnosed in the first year of life, is rare, with an incidence rate of 39.9 per million in the United States (Linabery and Ross, 2008). Approximately 60% of infant acute myeloid leukaemias (AML) and 80% of infant acute lymphoblastic leukaemias (ALL) involve rearrangement of the *MLL* gene (Ross and Spector, 2006), which are rare in older children and adults. Evidence of an *in utero* origin for some infant leukaemia comes from molecular studies, in which *MLL* translocations identified in leukaemia cells at diagnosis were also found in Guthrie cards collected shortly after birth (Gale *et al*, 1997; Greaves and Wiemels, 2003). Further, studies of twins with leukaemia revealed the presence of identical *MLL* rearrangements, suggesting that the initiating event occurs *in utero* in one twin and is transferred to the other twin via shared placental circulation (Greaves *et al*, 2003). The only firmly established risk factors for childhood leukaemia are *in utero* exposure to X-rays, specific genetic syndromes, such as Down's syndrome, and high birth weight (reviewed in Ross and Spector (2006)), which together explain only a very small proportion of leukaemia cases.

Regular use of aspirin or other non-steroidal anti-inflammatory drugs (NSAIDs) has been associated with a reduced risk of many adult cancers (Gonzalez-Perez *et al*, 2003; Bosetti *et al*, 2006; Elwood *et al*, 2009). Two studies of adult leukaemia suggested a protective effect with aspirin (Kasum *et al*, 2003; Weiss *et al*, 2006), but no effect with non-aspirin NSAIDs, and a possible increased risk with acetaminophen (Weiss *et al*, 2006). For childhood leukaemia, results are inconsistent with regard to maternal analgesic use (Wen *et al*, 2002; Shaw *et al*, 2004; Schuz *et al*, 2007), although many of these drugs are known to cross the placenta (Alano *et al*, 2001).

Given the paucity of risk factors identified for infant leukaemia, the strong evidence of an *in utero* origin, and the potential for possible effects on the developing fetus, we examined non-prescription maternal analgesic use and the risk of infant leukaemia.

## Materials and methods

Details for recruitment and data collection have been reported previously (Spector *et al*, 2005; Puumala *et al*, 2009, 2010). Cases comprised infants diagnosed with or treated for ALL or AML in the first year of life at one of the Children's Oncology Group (COG) institutions in the United States or Canada between January 1996 and October 2002 (phase I) and January 2003 and December 2006 (phase II). Additional eligibility criteria included the availability of a Spanish-speaking (phase II only) or English-speaking biological mother for telephone interview, and the absence of Down's syndrome. During phase I of recruitment, 348 cases were confirmed as eligible and interviews were completed for 240 (69%). For phase II, 345 potentially eligible cases were identified and 203 (59%) completed interviews.

During phase I of recruitment, controls were identified using random digit dialling, and frequency matched to cases on year of birth. Interviews were completed for 254 of 430 potentially eligible controls (59% field response rate). In phase II, controls were identified through a sample of state birth registries. Controls were frequency matched to cases on year of birth and region of residence based on the distribution of cases enrolled during phase I. Interviews were completed for 71 out of 267 potentially eligible controls (27% field response rate). The two control groups were found to be similar based on both maternal and infant characteristics (Puumala *et al*, 2009), and thus were combined into one control group for this analysis.

Informed consent was obtained from all participants. Information on maternal and infant characteristics was collected through computer-assisted telephone interviews with the biological mother. Analgesic questions included maternal use of aspirin, acetaminophen and non-aspirin NSAIDs, both before and after knowledge of pregnancy, including the frequency of use during these two time periods. The frequency of use categories before knowledge of pregnancy included fewer than 5 times, 5–25 times, and more than 25 times. For the period after knowledge of pregnancy through the end of the pregnancy, the categories were less than 10 times, 10–50 times, and greater than 50 times. Detailed reports on leukaemia cell cytogenetics and molecular abnormalities were obtained for cases and included leukaemia subtype (ALL, AML) and *MLL* gene translocation status (*MLL*+, *MLL*−, undetermined). Data on analgesic use during pregnancy were missing for two cases and one control. Few women reported using any type of analgesic more than 25 times before knowledge of pregnancy or more than 50 times after knowledge of pregnancy, which precluded examining frequency of use. Instead, for each type of analgesic, we created two variables: one for any use during the specified period and a second to indicate regular use (more than 5 times before knowledge of pregnancy or more than 10 times after knowledge of pregnancy).

Statistical analyses were carried out using SAS version 9.2 (SAS Institute, Cary, NC, USA). The possible association of analgesic use and the risk of infant acute leukaemia was evaluated using unconditional logistic regression. Odds ratios (OR) and 95% confidence intervals (95% CI) were calculated. All models were adjusted for the matching factor, year of birth. In addition, maternal characteristics listed in Table 1 were evaluated as potential confounders and included in the final regression model if they materially altered any of the analgesic variable estimates. All statistical tests were two-sided. Analyses were conducted separately by leukaemia subtype (ALL, AML), and *MLL* status (*MLL*+, *MLL*−).

## Results

Selected characteristics of 434 infant leukaemia cases (262 ALL, 172 AML) and 323 controls are shown in Table 1. Cases and controls were similar with respect to previous fetal loss, smoking during pregnancy, pre-pregnancy BMI, and infant gender and birth weight. However, compared with mothers of controls, mothers of cases were more likely to be younger (ALL), of Hispanic ethnicity (ALL, AML), of lower income (ALL, AML), and to report morning sickness (ALL). Case mothers were less likely to have completed education beyond high school (ALL), initiated multivitamin use before knowledge of pregnancy (ALL), reported alcohol consumption during pregnancy (AML), and confirmed pregnancy before the third month (AML).

We also evaluated the distribution of cases and controls with regard to analgesic use across selected potential confounders for the two pregnancy periods (data not shown). Before knowledge of pregnancy, acetaminophen was the most commonly (63%) used analgesic, followed by non-aspirin NSAIDs (31%) and aspirin (9%). Compared with non-regular users (⩽5 times), regular users were more likely to smoke during pregnancy and less likely to have completed education beyond high school or to report an annual household income greater than $75 000. Regular aspirin or non-aspirin NSAID users were more likely to be Caucasian and to drink during pregnancy, whereas regular acetaminophen users were less likely to use multivitamins before knowledge of pregnancy, compared with non-regular users. After knowledge of pregnancy, acetaminophen use increased (69%), whereas the use of aspirin and non-aspirin NSAIDs decreased (3 and 9%, respectively); only 19 case mothers and 13 control mothers used aspirin or non-aspirin NSAIDs regularly (⩾10 times).

Possible associations between infant leukaemia and maternal analgesic use, assessed both before and after knowledge of pregnancy, are shown in Table 2. There was no association between any use or regular use of aspirin, non-aspirin NSAIDs or acetaminophen with infant ALL, in either pregnancy period. However, for AML, there was a statistically significant or borderline significant reduced risk associated with any use of non-aspirin NSAIDs (OR=0.60, CI: 0.37–0.97) and acetaminophen (OR=0.66, CI: 0.43–1.01), respectively, but not with aspirin (OR=0.55, CI: 0.24–1.26); regular use of each analgesic did not reflect further reduction in risk. These inverse associations were observed only before knowledge of pregnancy, whereas they were attenuated toward the null after knowledge of pregnancy.

We also examined whether associations were confined to particular *MLL* groups of ALL and AML (data not shown). Among ALL cases, 155 were *MLL*+, 77 *MLL*−, and 30 with undetermined *MLL* status, whereas among AML cases there were 68 *MLL*+, 66 *MLL*−, and 38 undetermined. For AML, the inverse association observed for non-aspirin NSAIDs and acetaminophen was confined to cases that did not have the *MLL* gene rearrangement (OR=0.42, CI: 0.20–0.88, and OR=0.57, CI: 0.32–1.02, respectively), whereas ALL showed no association for either *MLL* subgroup.

## Discussion

We evaluated the association between maternal analgesic use and the risk of infant leukaemia. Although our results suggest that acetaminophen and non-aspirin NSAID use may be inversely associated with infant AML when taken early in pregnancy, it should be noted that these estimates were attenuated when confined to regular use (⩾5 times). The inverse association was confined to the subgroup containing no *MLL* gene rearrangement. This subtype most closely resembles childhood AML diagnosed after the age of 1 year, when only 5% of AML cases present with *MLL* gene rearrangements (Taki *et al*, 1996). We found no association between any of the analgesics studied and ALL risk.

Aspirin use had no effect on infant leukaemia risk in either pregnancy period. However, aspirin is not recommended during pregnancy, especially during the last trimester, although 10–30% of women may use aspirin in the first trimester before they are aware of their pregnancy (Werler *et al*, 1989; Kozer *et al*, 2002). In our study, aspirin was used by 10.5% of controls and 8.5% of cases before knowledge of pregnancy, and by 3.7% of controls and 3.3% of cases after knowledge of pregnancy.

Two out of three studies found no association between maternal analgesic use and risk of leukaemia in children 0–14 years of age (Wen *et al*, 2002; Schuz *et al*, 2007). In contrast, one study reported a borderline significant two-fold increased risk of childhood ALL associated with prescription or over-the-counter anti-inflammatory drug use during pregnancy (Shaw *et al*, 2004). These three studies, however, included few women who used these medications during pregnancy. In addition, associations may have been diluted owing to broad medication groupings and few details on specific medications used.

Strengths and weaknesses of our study are relevant. This is the largest infant leukaemia study to date and included availability of *MLL* gene status. Identification of cases through COG provided a nearly population-based study population, as COG institutions see ∼100% of leukaemia cases aged 0–4 years (Ross *et al*, 1996). We evaluated different classes of analgesics, but not their doses. The small sample size for some subgroup analyses warrants caution in interpretation of the relevant associations. In particular, given the low frequency of aspirin use, especially after knowledge of pregnancy, we had limited power to evaluate aspirin effects. Recall bias is a concern in case–control studies, but it is unlikely that mothers with ALL infants would recall their exposures differently from mothers with AML. Further, the early age of onset helped to limit the recall period. There is also some concern with regard to selection bias. We found that use of analgesics varied by race, income, educational attainment, and smoking or alcohol consumption during pregnancy. Although we adjusted for maternal race, ethnicity, income, and alcohol consumption during pregnancy, residual confounding may remain.

In summary, this study found no significant association between the use of over-the-counter analgesics and risk of infant leukaemia.

## Figures and Tables

**Table 1:** Selected characteristics according to infant leukaemia status^a^

	**Controls (*n*=323)**	**Acute llymphoblastic leukaemia (*n*=262)**			**Acute myeloid leukaemia (*n*=172)**		
	**No. (%)**	**No. (%)**	**OR**	**95% CI**	**No. (%)**	**OR**	**95% CI**
*Maternal characteristics*							
Age at index child's birth							
15–24	57 (18)	67 (26)	1.54	(0.97–2.46)	44 (26)	2.59	(1.46–4.60)
25–29	114 (35)	86 (33)	1.00	Reference	35 (20)	1.00	Reference
30–34	94 (29)	73 (28)	1.08	(0.70–1.66)	60 (35)	2.22	(1.32–3.75)
35–45	58 (18)	36 (14)	0.85	(0.51–1.43)	32 (19)	2.01	(1.10–3.67)
Education							
⩽High school	91 (28)	93 (36)	1.00	Reference	53 (31)	1.00	Reference
>High school	232 (72)	169 (65)	0.68	(0.47–0.98)	119 (69)	0.80	(0.52–1.23)
Ethnicity							
Caucasian	273 (85)	199 (76)	1.00	Reference	130 (76)	1.00	Reference
African American	18 (6)	11 (4)	0.67	(0.30–1.51)	7 (4)	0.88	(0.34–2.27)
Hispanic	15 (5)	29 (11)	2.60	(1.32–5.09)	24 (14)	2.51	(1.22–5.15)
Other	17 (5)	23 (9)	1.93	(0.98–3.81)	11 (6)	1.30	(0.57–2.98)
Household income							
⩽$30 000	95 (30)	99 (38)	1.51	(1.02–2.24)	54 (32)	1.01	(0.64–1.60)
$30 001–$75 000	145 (45)	105 (40)	1.00	Reference	82 (48)	1.00	Reference
>75 000	81 (25)	57 (22)	0.82	(0.53–1.27)	34 (20)	0.57	(0.34–0.96)
Pre-pregnancy BMI							
Under/normal wt.	192 (59)	143 (55)	1.00	Reference	92 (53)	1.00	Reference
Overweight/obese	131 (41)	117 (45)	1.13	(0.80–1.59)	80 (47)	1.15	(0.77–1.70)
Weeks pregnant when confirmed							
1–4	94 (29)	89 (34)	1.00	Reference	59 (34)	1.00	Reference
5–8	179 (55)	136 (52)	0.73	(0.50–1.07)	83 (48)	0.60	(0.39–0.95)
⩾9	50 (15)	37 (14)	0.71	(0.41–1.21)	30 (17)	0.86	(0.48–1.56)
Morning sickness							
No	119 (37)	75 (29)	1.00	Reference	51 (30)	1.00	Reference
Yes	204 (63)	187 (71)	1.42	(0.99–2.04)	121 (70)	1.40	(0.92–2.13)
Previous fetal loss							
No	240 (74)	197 (75)	1.00	Reference	133 (77)	1.00	Reference
Yes	83 (26)	65 (25)	1.11	(0.75–1.64)	39 (23)	1.00	(0.63–1.58)
Smoking during pregnancy							
No	258 (80)	212 (81)	1.00	Reference	149 (87)	1.00	Reference
Yes	65 (20)	50 (19)	1.03	(0.67–1.58)	23 (13)	0.73	(0.43–1.24)
Drinking during pregnancy							
No	254 (79)	219 (84)	1.00	Reference	151 (89)	1.00	Reference
Yes	69 (21)	43 (16)	0.74	(0.48–1.14)	19 (11)	0.48	(0.27–0.84)
Multivitamin use before knowledge of pregnancy							
No	140 (43)	135 (52)	1.00	Reference	82 (48)	1.00	Reference
Yes	183 (57)	127 (48)	0.71	(0.51–1.00)	90 (52)	0.79	(0.53–1.17)
							
*Child characteristics*							
Gender							
Male	155 (48)	131 (50)	1.00	Reference	84 (49)	1.00	Reference
Female	168 (52)	131 (50)	0.95	(0.68–1.34)	88 (51)	0.99	(0.67–1.47)
Birth weight (g)							
<2500	17 (5)	9 (3)	0.63	(0.26–1.49)	14 (8)	1.28	(0.58–2.84)
2500–4000	257 (80)	208 (79)	1.00	Reference	135 (78)	1.00	Reference
>4000	49 (15)	45 (17)	1.22	(0.77–1.94)	23 (13)	0.87	(0.50–1.53)
Firstborn							
No	205 (63)	138 (53)	1.00	Reference	109 (63)	1.00	Reference
Yes	118 (37)	124 (47)	1.45	(1.03–2.04)	63 (37)	0.88	(0.58–1.32)

Abbreviations: CI=confidence interval; OR=odds ratio.

a Logistic regression models adjusted for the matching factor and index child's year of birth (continuous). Values may not sum to the total number of cases and controls owing to missing values.

**Table 2:** Association of maternal pain reliever use during pregnancy and the risk of infant leukaemia^a^

		**Acute lymphoblastic leukaemia**			**Acute myeloid leukaemia**		
	***N* (%)**	***N* (%)**	**OR**	**95% CI**	***N* (%)**	**OR**	**95% CI**
*Aspirin*							
Before knowledge of pregnancy							
Any use							
No	287 (89.4)	236 (90.4)	1.00	Reference	158 (94.6)	1.00	Reference
Yes	34 (10.6)	25 (9.6)	1.03	0.58–1.85	9 (5.4)	0.55	0.24–1.26
Regular use^b^							
No	308 (96.3)	253 (96.9)	1.00	Reference	163 (97.6)	1.00	Reference
Yes	12 (3.8)	8 (3.1)	1.25	0.48–3.31	4 (2.4)	0.80	0.22–2.97
After knowledge of pregnancy							
Any use							
No	309 (96.3)	252 (96.6)	1.00	Reference	163 (97.0)	1.00	Reference
Yes	12 (3.7)	9 (3.5)	1.21	0.48–3.05	5 (3.0)	0.96	0.32–2.92
Regular use^b^							
No	314 (97.8)	255 (97.7)	1.00	Reference	166 (98.8)	1.00	Reference
Yes	7 (2.2)	6 (2.3)	1.55	0.49–4.97	2 (1.2)	0.69	0.13–3.56
							
*Non-aspirin NSAIDS*							
Before knowledge of pregnancy							
Any use							
No	219 (68.2)	168 (64.9)	1.00	Reference	130 (77.4)	1.00	Reference
Yes	102 (31.8)	91 (35.1)	1.15	0.80–1.67	38 (22.6)	0.60	0.37–0.97
Regular use^b^							
No	293 (91.3)	231 (89.2)	1.00	Reference	158 (94.1)	1.00	Reference
Yes	28 (8.7)	28 (10.8)	1.41	0.78–2.57	10 (6.0)	0.68	0.29–1.59
After knowledge of pregnancy							
Any use							
No	292 (91.0)	232 (88.9)	1.00	Reference	158 (94.1)	1.00	Reference
Yes	29 (9.0)	29 (11.1)	1.33	0.75–2.37	10 (6.0)	0.81	0.36–1.83
Regular use^b^							
No	315 (98.1)	252 (96.6)	1.00	Reference	166 (98.8)	1.00	Reference
Yes	6 (1.9)	9 (3.5)	1.94	0.64–5.93	2 (1.2)	0.73	0.13–4.02
							
*Acetaminophen*							
Before knowledge of pregnancy							
Any use							
No	113 (35.2)	92 (35.3)	1.00	Reference	76 (45.2)	1.00	Reference
Yes	208 (64.8)	169 (64.8)	1.16	0.80–1.68	92 (54.8)	0.66	0.43–1.01
Regular use^b^							
No	268 (83.5)	215 (82.4)	1.00	Reference	146 (86.9)	1.00	Reference
Yes	53 (16.5)	46 (17.6)	1.11	0.70–1.78	22 (13.1)	0.83	0.45–1.52
After knowledge of pregnancy							
Any use							
No	94 (29.4)	80 (30.7)	1.00	Reference	57 (33.9)	1.00	Reference
Yes	226 (70.6)	181 (69.4)	1.03	0.70–1.53	111 (66.1)	0.79	0.50–1.24
Regular use^b^							
No	247 (77.2)	191 (73.2)	1.00	Reference	137 (81.6)	1.00	Reference
Yes	73 (22.8)	70 (26.8)	1.27	0.85–1.90	31 (18.5)	0.77	0.46–1.30

Abbreviations: CI=confidence interval; NSAIDs=non-steroidal anti-inflammatory drugs; OR=odds ratio.

a Logistic regression models are adjusted for maternal age (15–24, 25–29, 30–34, 35–45), race (white, black, Hispanic, other), alcohol consumption during pregnancy (yes/no), household income (<$30 000, $30 001–$75 000, >$75 000), and index child's year of birth (continuous).

b Regular use of a pain reliever before and after knowledge of pregnancy is defined as ⩾5 and ⩾10 times, respectively.
